# Conformational coupling by trans-phosphorylation in calcium calmodulin dependent kinase II

**DOI:** 10.1371/journal.pcbi.1006796

**Published:** 2019-05-31

**Authors:** Alessandro Pandini, Howard Schulman, Shahid Khan

**Affiliations:** 1 Department of Computer Science—Synthetic Biology Theme, Brunel University London, Uxbridge, United Kingdom; 2 The Thomas Young Centre for Theory and Simulation of Materials, London, United Kingdom; 3 Computational Cell and Molecular Biology, the Francis Crick Institute, London, United Kingdom; 4 Allosteros Therapeutics Inc., Sunnyvale, CA, United States of America; 5 Molecular Biology Consortium, Lawrence Berkeley National Laboratory, Berkeley, CA, United States of America; Max Planck Institute for Biophysical Chemistry, GERMANY

## Abstract

The calcium calmodulin-dependent protein kinase II (CaMKII) is a dodecameric holoenzyme important for encoding memory. Its activation, triggered by binding of calcium-calmodulin, persists autonomously after calmodulin dissociation. One (receiver) kinase captures and subsequently phosphorylates the regulatory domain peptide of a donor kinase forming a chained dimer as the first stage of autonomous activation. Protein dynamics simulations examined the conformational changes triggered by dimer formation and phosphorylation, aimed to provide a molecular rationale for human mutations that result in learning disabilities. Ensembles generated from X-ray crystal structures were characterized by network centrality and community analysis. Mutual information related collective motions to local fragment dynamics encoded with a structural alphabet. Implicit solvent tCONCOORD conformational ensembles revealed the dynamic architecture of inactive kinase domains was co-opted in the activated dimer but the network hub shifted from the nucleotide binding cleft to the captured peptide. Explicit solvent molecular dynamics (MD) showed nucleotide and substrate binding determinants formed coupled nodes in long-range signal relays between regulatory peptides in the dimer. Strain in the extended captured peptide was balanced by reduced flexibility of the receiver kinase C-lobe core. The relays were organized around a hydrophobic patch between the captured peptide and a key binding helix. The human mutations aligned along the relays. Thus, these mutations could disrupt the allosteric network alternatively, or in addition, to altered binding affinities. Non-binding protein sectors distant from the binding sites mediated the allosteric signalling; providing possible targets for inhibitor design. Phosphorylation of the peptide modulated the dielectric of its binding pocket to strengthen the patch, non-binding sectors, domain interface and temporal correlations between parallel relays. These results provide the molecular details underlying the reported positive kinase cooperativity to enrich the discussion on how autonomous activation by phosphorylation leads to long-term behavioural effects.

## Author summary

Protein kinases play central roles in intracellular signalling. Auto-phosphorylation by bound nucleotide typically precedes phosphate transfer to multiple substrates. Protein conformational changes are central to kinase function, altering binding affinities to change cellular location and shunt from one signal pathway to another. In the brain, the multi-subunit kinase, CaMKII is activated by calcium-calmodulin upon calcium jumps produced by synaptic stimulation. Auto-transphosphorylation of a regulatory peptide enables the kinase to remain activated and mediate long-term behavioural effects after return to basal calcium levels. A database of mutated residues responsible for these effects is difficult to reconcile solely with impaired nucleotide or substrate binding. Therefore, we have computationally generated interaction networks to map the conformational plasticity of the kinase domains where most mutations localize. The network generated from the atomic structure of a phosphorylated dimer resolves protein sectors based on their collective motions. The sectors link nucleotide and substrate binding sites in self-reinforcing relays between regulatory peptides. The self-reinforcement is strengthened by phosphorylation consistent with the reported positive cooperativity of kinase activity with calcium-calmodulin concentration. The network gives a better match with the mutations and, in addition, reveals target sites for drug development.

## Introduction

The calcium calmodulin-dependent protein kinase (CaMKII) is a multifunctional, multi-subunit eukaryotic protein kinase (EPK). It has key roles in calcium regulation of neuronal and cardiovascular physiology [1–3]. EPKs mediate reactions whose malfunction promotes disease across a broad physiologic spectrum. The canonical EPK has a distinctive bi-lobed structure that exploits diverse strategies to achieve allosteric regulation [4].

CaMKII has a canonical kinase domain (KD) tethered via a linker to an equally well-conserved association domain (AD) that forms a central hub of the dodecameric holoenzyme with two hexamer rings that stack with mirror symmetry. Linker diversity generates isoforms and splice variants. The kinase is activated by rises in cellular calcium that enable calcium-calmodulin (Ca^2+^/CaM) to bind to and displace an autoinhibitory regulatory domain. The autoinhibitory domain occludes interaction of CaMKII with anchoring proteins, such as NMDA receptor subunit GluN2B [5, 6]. More subunits are activated at an increasing frequency of calcium pulse trains. The tuning frequency depends on the switching kinetics between the holoenzyme open and closed states [7].

In the closed state, the substrate binding surface is occluded by an auto-inhibitory segment composed of an N-terminal segment (R1) and C-terminal α-helices (R2, R3) and ATP affinity is low [8]. R1 contains the primary auto-phosphorylation site (T286 in α subunit, T287 in others), as well as residues for O-GlcNAC modification (S279) and oxidation (M281, M282, C290 (δ isoform)). R2 has a CaM recognition motif. Ca^2+^/CaM binding displaces the regulatory segment to switch CaMKII to the open state, followed by segment capture and T286 autophosphorylation by an adjacent, open (activated) subunit [8, 9] in the holoenzyme. R3 has additional threonine residues (T305, T306) that are primarily phosphorylated when CaM dissociates from a Ca^2+^-independent (autonomous) kinase [10, 11] to counteract T286 phosphorylation [12]. X-ray crystal structures show that R1 and its binding determinants in the KD core adopt different conformations. Electron spin resonance (ESR) reports that R1 becomes unstructured upon T286 phosphorylation, while R2 and R3 are disordered in solution [13].

Persistent CaMKII activity autonomous of Ca^2+^/CaM underlies conversion of transient synaptic stimulation to long-term potentiation (LTP); a fundamental problem of neuronal CaMKII biochemistry that underlies certain forms of learning and memory [6, 14–17]. Recent studies have identified more than a dozen human mutations in CaMKII that result in various degrees of learning disabilities [18, 19]. These mutations, when mapped onto structure, localize largely with R1 or mutations that alter KD interactions with substrates rather than R2. The previously described binding mutations form S (substrate binding) and T (Thr286 docking) sites [20, 21]). The substrate binding determinants overlap with the R1 binding groove that can be partitioned into A, B and C sites [8, 22]. The T-site is eliminated upon release of the autoinhibitory segment by rotation of helix αD whose movement helps form the B site [23]. An outstanding issue is whether the human mutations act independently or as part of a collective network to disrupt allosteric communication responsible for frequency tuning [24]. The conventional form of autonomous activity follows T286 trans-auto-phosphorylation, which maintains activity after dissociation of Ca^2+^/CaM. Structures of CaMKII complexes with R1 regulatory segments of one subunit (“donor”) captured by the adjacent subunit (“receiver”) upon Ca^2+^/CaM binding, give important snapshots into trans-phosphorylation [8, 9]. A hydrophobic clamp of helix αD residues within the receiver KD captures the R1 in an interaction that propagates throughout the crystal lattice.

Here, we used tCONCOORD, based on stochastic distance constraints [25], and explicit solvent molecular dynamics (MD) as complementary methods to address the mechanistic basis underlying the medical phenotypes of the human mutations. tCONCOORD ensembles from KD structures described collective motions and conformational coupling between the nucleotide and substrate binding sites. MD examined fine-grained architectural modulations due to primary site phosphorylation on the long-range allosteric network to establish an analytical framework for the coupling within and between subunits. We characterized the long-range allosteric relays in a KD dimer extracted in silico from the crystal lattice as well as KD monomer structures. Two strategies analyzed the network architecture based on mutual information. First, the centrality profile of the complete network of local correlated motions was determined to identify relays of the most strongly coupled fragments. Second, the protein was partitioned into contiguous sectors, with coupled dynamics, that act as semi-rigid bodies–henceforth defined as “communities” [26]. Community size and cross-talk tracked the transition between the auto-inhibited and phosphorylated states.

We show that the captured R1 acts as a spring, rather than a “grappling hook” [8] to couple nucleotide and substrate binding sites [27, 28]. The capture freezes out C-lobe core motions to generate long-distance signal relays between R1 segments of adjacent subunits. The human mutations align along the relays. T286 phosphorylation strengthens the relays to suggest how it might facilitate the inter-subunit conformational spread and modulate affinities at distant sites for calmodulin [29] and cytoskeletal actin [30]. Notably, we identify a hitherto uncharacterized sector to guide the design of allosteric inhibitors. The simulations should complement experimental ESR [13] and FRET measurements [31, 32] on activated CaMKII dimers for dissection of the activation mechanism.

## Results

### The human mutations localize to the KD C-lobe and R1

**Fig 1:**
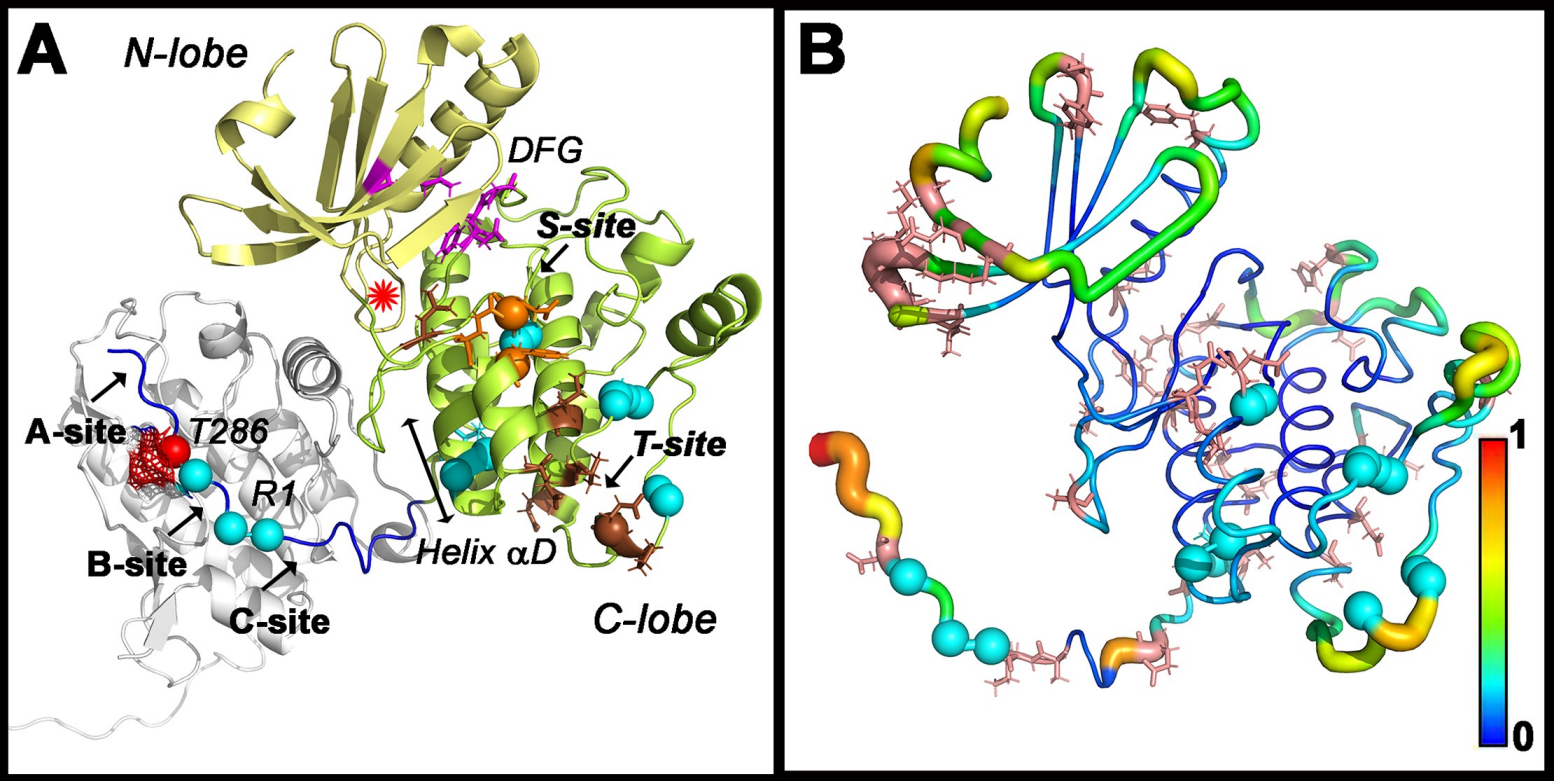
CaMKII Functional Mutations. Spheres (cyan) show residue positions where mutations cause learning disabilities in humans. **A. CaMKII KD architecture.** Structure of the CaMKII kinase domain (3KK8) with N-terminal (pale yellow) and C-terminal (lemon) lobes and R1 regulatory segment (blue). Spheres mark S (orange) and T site (brown) binding mutations [20], primary auto-phosphorylation site (T286 (red with mesh)). Helix αD (double arrow bar). R1 is bound to a groove in an adjacent subunit (white)). The groove has three sites–A, B and C. The asterisk (red) marks the magnesium ion location. **B. CaMKII KD flexibility and sequence variation.** RMSF profile computed from the 3KK8 ensemble. Stick (salmon red) representations identify variable residue positions as identified from the MSA (S1 Fig). Vertical bar indicates color scale (1 = high (red); 0 = low (blue)).

**Fig 1A** provides a road-map of the architectural elements investigated in this study. The R1 with T286 binds to a groove within the adjacent receiver KD C-lobe that overlaps with the receiver’s S-site and has been sub-divided into three pockets A, B and C [8] in the nematode *C*.*elegans* KD dimer (PDB ID:3KK8)). The A-site forms a canonical substrate docking interaction with the ATP binding cleft that contains the DFG motif (D_156_FG_158_); the B-site forms a hydrophobic patch with helix αD; the C-site has acidic residues for salt-bridges with R1. Residue positions with mutations in the human homolog that result in learning disabilities as well as S-site and T-site mutations are mapped onto the donor KD. The human mutations localized to R1 (I272, H282, R284, T286), or overlapped with, or were adjacent to the S and T-sites (F98S, E109, P138). However, a significant fraction mapped elsewhere.

Variable residue positions, identified from the multiple sequence alignment (MSA) of > 500 KD sequences, localized to surface accessible loops when mapped onto 3KK8. The MSA shows strong sequence conservation between species and minimal differences between isoforms within species. The phylogenetic tree indicates that nematode and human sequences are among the most distantly related (**S1A and S1B Fig)**. Secondary structure predicted from the MSA for the *C*. *elegans*, rat and human sequences matched that observed in the crystal structure. The predictions back ESR evidence for R2 and R3 disorder [13]. The human mutations localized to mainly conserved residue positions in both rigid and flexible segments of the KD C-lobe and R1.

The flexibility profile recorded by the root mean square fluctuations (RMSF) of residue positions computed from the tCONCOORD ensembles of 3KK8 dimers (**Fig 1B)** is largely consistent with the B-factor values in the crystal structure with differences due, in part, from crystal contacts. **(S1C Fig).** There are also differences between donor and receiver domains that are considered below in detail.

### The captured R1 forms the central node of the allosteric network

**Fig 2:**
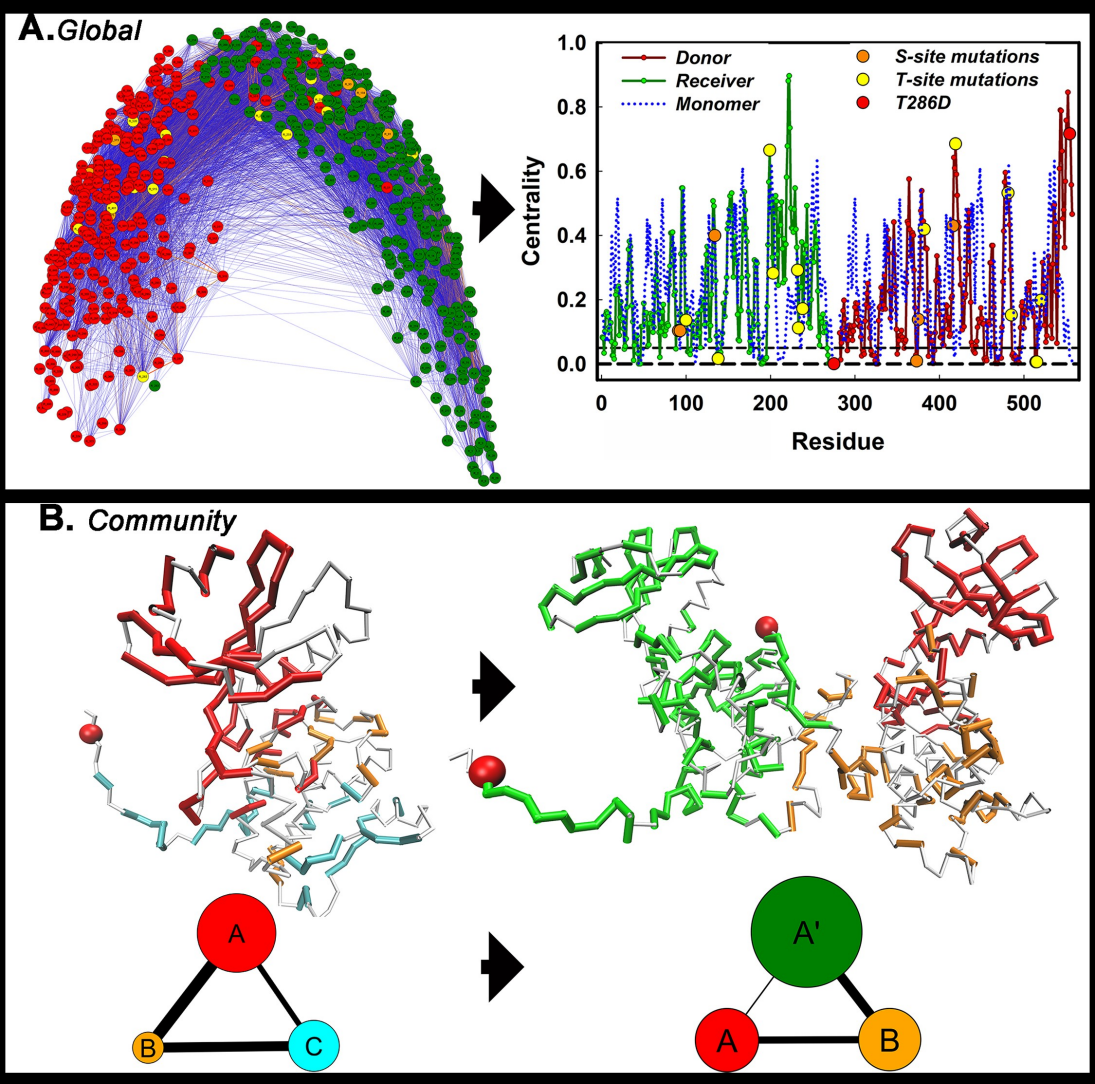
Adaptation of the dynamic global and community architecture upon dimerization. **A.** The global network (donor KD (red), receiver KD (green) and its eigenvector centrality. Spheres mark S (orange) and T site (yellow) mutant residue positions and TPO286 (red). **B.** Community members of the CaMKII KD (KK8) monomer (open-form) and dimer (ribbon representations) mapped onto the structures with associated community graphs (node diameter = community size; edge thickness = coupling strength) below.

We analyzed local protein dynamics to decipher allosteric signal propagation [33–35]. The 3KK8 dimer was extracted from the crystal structure electron density with the R1 of the donor KD captured (“bound”) by the adjacent receiver; while the R1 of the receiver KD was unconstrained (“free”), immersed in the solvent. Three, separate, explicit solvent MD replica runs of the 1.7-angstrom resolution structure of the phosphorylated 3KK8 dimer and its non-phosphorylated derivative was performed. Four-residue fragments were encoded by the structural alphabet (SA) [36] in structures within the conformational ensembles and trajectories. In the network, the nodes were the fragments (n = 560), while edges were the normalized mutual information (nMI) filtered set of possible correlations (n*(n-1)/2) = 156520) (Methods). The map reflected the contribution of individual fragments to the dynamics and is optimal for detection of network nodes (**Fig 2A**). The T and S sites emerged as prominent nodes in addition to the captured R1 However, the network architecture is too dense to visualize the spatial extent or interaction strength of the connections formed by individual nodes.

**Fig 3:**
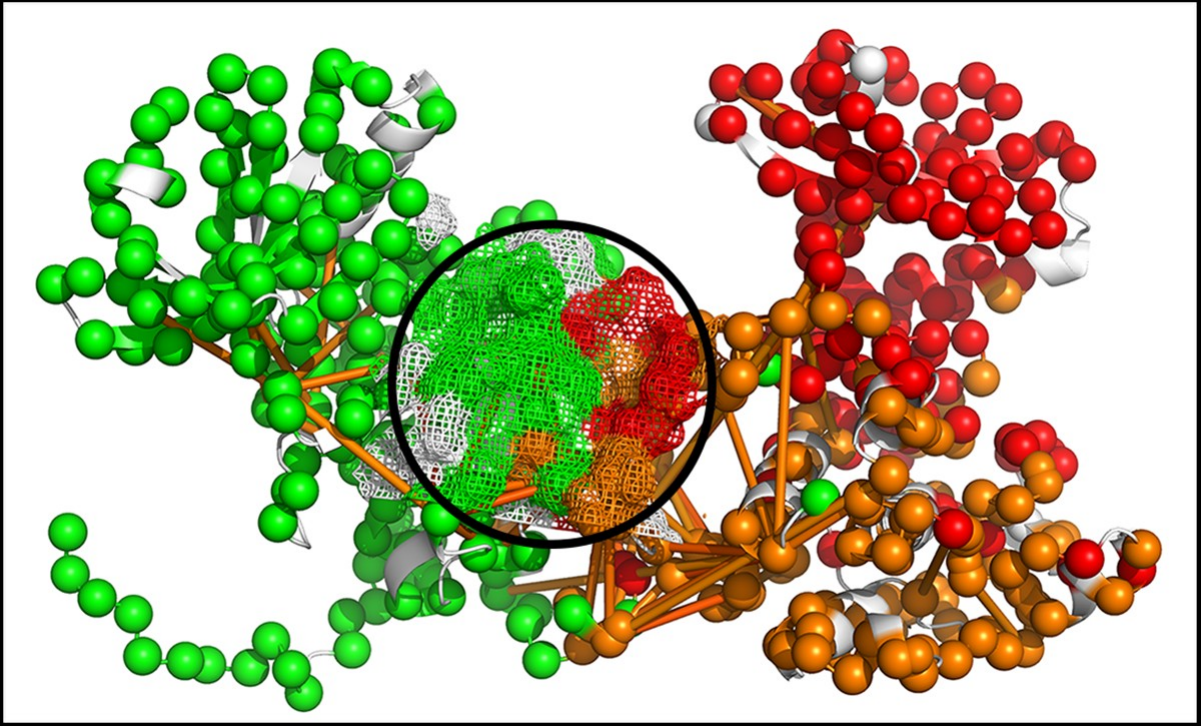
The captured R1 forms the community hub of the chained dimer. Distinct communities (color-coded C^α^ atoms, as in Fig 2B (spheres), and top fragment-fragment couplings (nMI > 0.2 (orange lines)) are superimposed onto the structure. Mesh (circled) marks the captured R1 and contacting residues.

Communities (n > 3), each represented as a node, greatly simplify network visualization of the connectivity by encoding concerted domain motions intermediate between global motions and residue-level RMSF fluctuations. Community membership and interaction strength were computed based on spectral decomposition [37, 38]. Our methodology reproduced the published community map of the PKA catalytic subunit (PDB ID: 1CMK) [26] and showed that the CaMKII KD had the similar dynamic architecture to PKA with the ATP binding site forming the community hub (**Fig 2B**, **S2 Fig**). Three major communities (A, B, C) converged at the ATP site. The N-lobe community A included the ATP binding cleft and part of the A-site. The C-lobe was split along its centre into community B with B-site helix αD and community C with the C-site acid residue pocket. Donor KD communities B and C coalesced with dimer formation (B’), while one community (A’) accounted for the receiver KD. The top couplings from the global network scored on nMI were superimposed on the community architecture (**Fig 3**). The captured R1 replaced the ATP binding site as the community hub. The couplings congregated around it to link the donor A site with the receiver B and C sites.

### The DFG motif and helix αD link communities in diverse KD structural states

**Fig 4:**
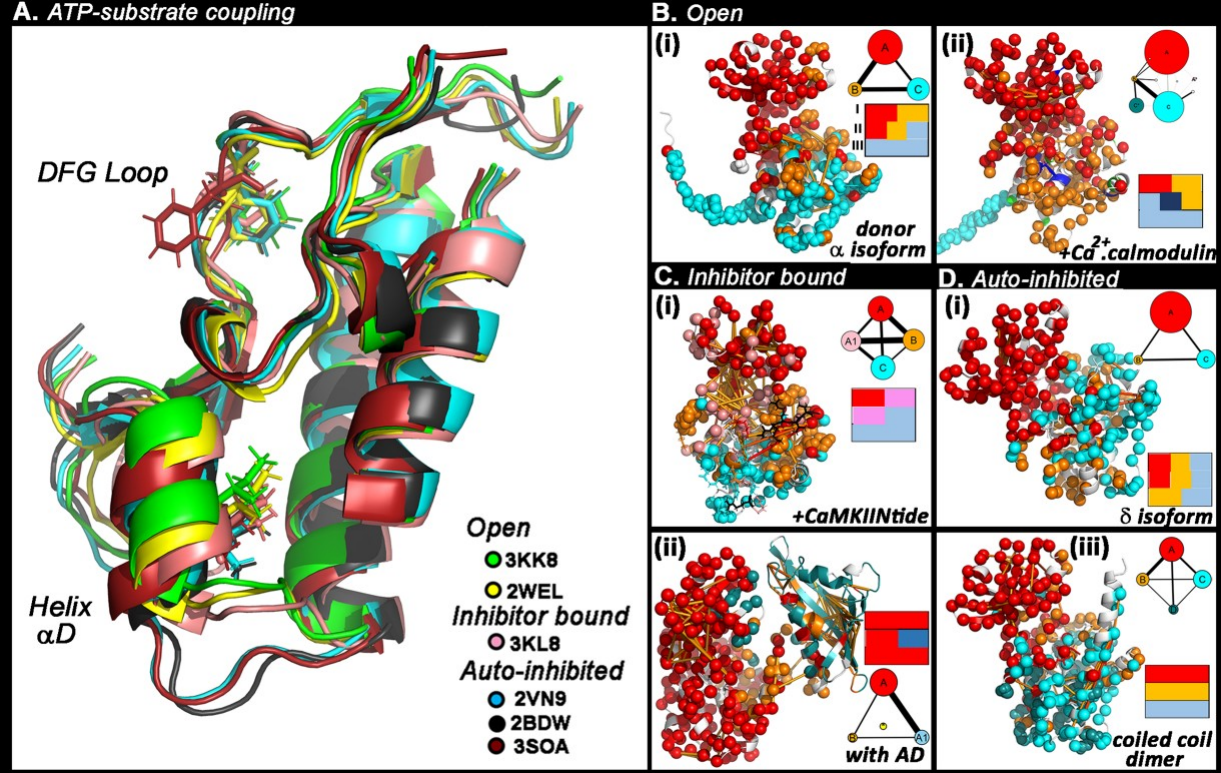
**A. Analysis of the coupling between ATP and substrate binding sites.** Aligned KD C-lobe crystal structures. RMSD’s (angstroms)– 2BDW (reference), 2VN9 (0.30), 2WEL (0.34), 3KK8 (0.23), 3KL8 (0.62), 3SOA (0.29). **Community dynamics in KD crystal structures**. Colours (red (N-lobe), orange (central C-lobe), cyan (basal C-lobe)) denote major communities following the 3KK8 donor KD community structure (Fig 2B). Top dynamic couplings (orange lines) are mapped on the structures as in Fig 3. **Insets:** Community members of the ATP binding motif D_156_FG_158_ **(I)**, helix αD L_97_FEDIVAR_104_ (**II**) and C site P_235_EWD_238_ (**III**) in **B—Open forms**. **(i).** Nematode CaMKII with free R1 (3KK8 donor KD), **(ii).** a human δ with bound calmodulin (2WEL). The segment bound to calmodulin forms a distinct community (navy) with community B partitioned into smaller communities. **C—Inhibitor-bound form. (i)** Nematode CaMKII with bound CaMKIINtide (3KL8). **D—Auto-inhibited forms**. **(i).** Human δisoform (2VN9). **(ii).** Complete (KD with AD) human CaMKII α (3SOA). Contacts between the N-lobe and AD form a distinct community (yellow). **(iii).** Nematode CaMKII stabilized by R2 helix (coiled-coil dimer) (2BDW).

We compared the 3KK8 KD with other CaMKII KD structural states in the Protein Data Bank (PDB) to first correlate community dynamics with monitors of kinase activity. Activation involves inward motion of the DFG side-chains for nucleotide binding and inward tilt of helix αD for contact with substrate peptides in many diverse EPKs [39]. The states could be grouped into three categories–open, inhibitor-bound and auto-inhibited. tCONCOORD conformational ensembles were generated. The dominant conformations for each ensemble were identified from cluster analysis and aligned (**Fig 4A**). The two open activated states (3KK8, 2WEL) and the inhibitor-bound form (3KL8) had inwardly oriented DFG motifs and αD helices. In contrast, the auto-inhibited states (2BDW, 2VN9, 3SOA) had outwardly rotated helix αDs. While 3SOA has an outwardly oriented DFG motif, the loops for 2BDW and 3SOA had inward orientation albeit at a position distinct from that assumed by activated states.

We next catalogued the community membership of the DFG motif (**I**), fragment L_97_FEDIVAR_104_ from B-site helix αD (**II**) and fragment P_235_EWD_238_ from the C-site acid pocket (**III**) (**Fig 4B–4D**). The open forms (**Fig 4B**) differed in their community architecture owing to the bound calmodulin. The calmodulin fragmented community B, with R1 dynamics uncoupled from the C-lobe. In both forms, fragments I and II had multiple memberships in communities A and B. The CaMKIINtide inhibitor peptide associated with sites A, B and C similarly to the captured R1 [8] (**Fig 4C**). Accordingly, community interactions are substantially increased, even though the communities did not coalesce. Community interactions were weak in the auto-inhibited states (human δ, human αfull-length subunit, and a KD extracted from the nematode coiled-coil dimer) (**Fig 4D**). Community A extended into the C-lobe when AD contacts limited the relative motions of the N and C-lobes to each other (3SOA). Fragment II connected the dominant communities as did fragment I in the auto-inhibited human δ, as well as the human α KD.

In conclusion, the coupled ATP binding DFG motif (Fragment I) and helix αD (Fragment II) form a universal hub in activated and inactivated CaMKII KD states based on their multiple community membership. The hub is robust to variations in community interaction strength. Inward movement of helix αD is diagnostic for activation, but that of the DFG motif not necessarily so.

### The captured R1 freezes KD core motions

We next characterized collective motions, extracted by principal component analysis (PCA) to better understand the monomer to dimer transition. The PCA projected the principal eigenvectors from different replica runs onto common principal axes, as outlined in Methods. Eigenvectors were also compared [40] for the principal motions (PCs) (**S3 Fig**). The overlap was comparable to that reported for similar size assemblies [40] consistent with confinement of the essential modes within a reproducible subspace [41].

**Fig 5:**
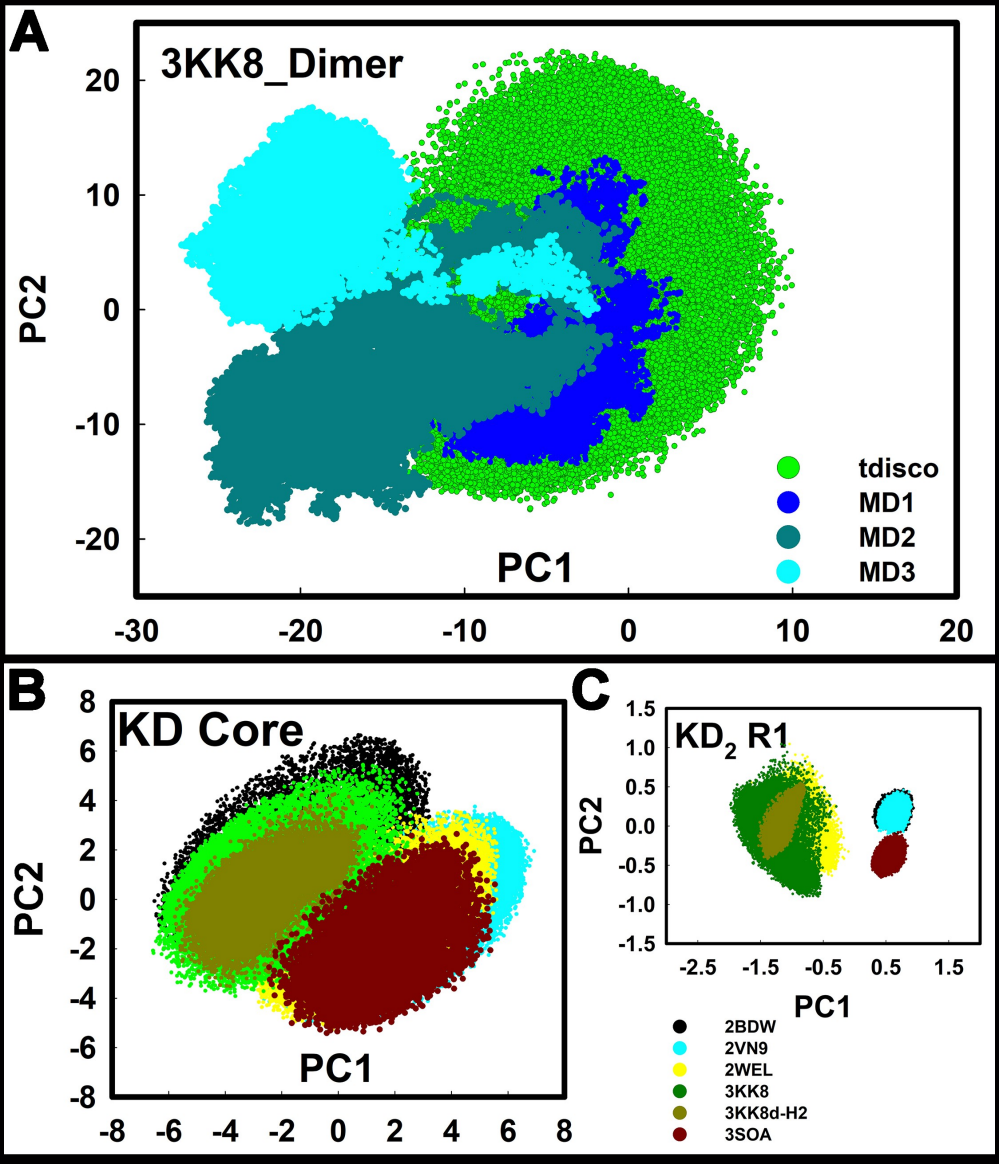
The captured R1 inhibits collective motions of the kinase core. PC1PC2 plots of tCONCOORD ensembles show the relation between the collective motions recorded by the amplitudes of the principal components PC1 and PC2 in CaMKII KD crystal structures. **A.** PC1PC2 plots for the 3KK8 dimer compare the tCONCOORD ensemble (tdisco) of the activated mutant (TPO286D) with MD replicas of the TP0 dimer. **B, C.** PC1PC2 plots of the (**B**) KD core (N-lobe + C-lobe minus R1) and (**C**) R1 of the donor (3KK8) and receiver (3KK8-H2) KDs of the 3KK8 dimer compared with plots for the KD from 4 crystal structures (auto-inhibited (2BDW) and CaM bound (2WEL) C. elegans; auto-inhibited human δ (2VN9) and α (3SOA) isoforms. Structure colour code follows 3A.

PC1PC2 plots compared the amplitudes of the dimer motions obtained by MD and tCONCOORD (**Fig 5A**). The subspace positional fluctuations along the first two PCs accounted for >70% of the total motion. The essential dynamics represented by the first ten PCs primarily delineated relative motions of N and C lobes (S3 Fig). The initial drift could result from Brownian motion and/or shallow local minima in the 3D energy landscape [42]. The PC1PC2 conformational space sampled during each MD run was typically greater 2/3 the space sampled by the tCONCOORD ensemble. The space sampled by the combined MD trajectories had considerable overlap (> 90%) with the ensemble.

Motions of the subunit KD cores and R1 segments were compared to evaluate the extent to which captured R1 motions determined dimer flexibility. The mechanical hinge and shear surface responsible for the PC motions formed community interfaces, with the ATP binding cleft as the hub in the monomer (**S1 Movie)**. The captured R1 was revealed to be the central hinge or hub for the dimer PC motions (**S2 Movie)**. The PCA shows that the captured R1 acts as an allosteric effector to freeze-out the motions of the kinase core.

Simulations on other CaMKII KD structures revealed the principal dimer motions were three-fold greater relative to monomer KD core motions. PC1PC2 spread of the receiver KD core was reduced (> 60%) relative to the spread of donor KD core and, indeed, KD cores from the other structures (**Fig 5B**). This result is consistent with the differences in computed B-factor values for the donor and receiver KDs (S1C Fig). The PC1PC2 plots for the R1 segment ensembles formed two distinct groups (**Fig 5C)**. The group with smaller spread represented auto-inhibited structures. The cluster with larger spread (4x) consisted of the captured R1 and free R1 segments. The PC1PC2 spread of the captured R1 was reduced by 50% relative to the free R1 segments. The scenario most simply consistent with these results is of a 3-state transition between autoinhibition and activation. R1 is immobile docked as pseudo-substrate (“auto-inhibited” state), mobile when free in solution (“open” state) and constrained upon subsequent capture (“activated” state). The mobility of the KD core does not change when R1 is displaced but it is notably reduced upon R1 capture. Therefore, R1 freezes KD core collective motions more effectively when captured than when docked as pseudo-substrate, but with a compensatory increase in its own flexibility.

### Energetics of the chained dimer

**Fig 6:**
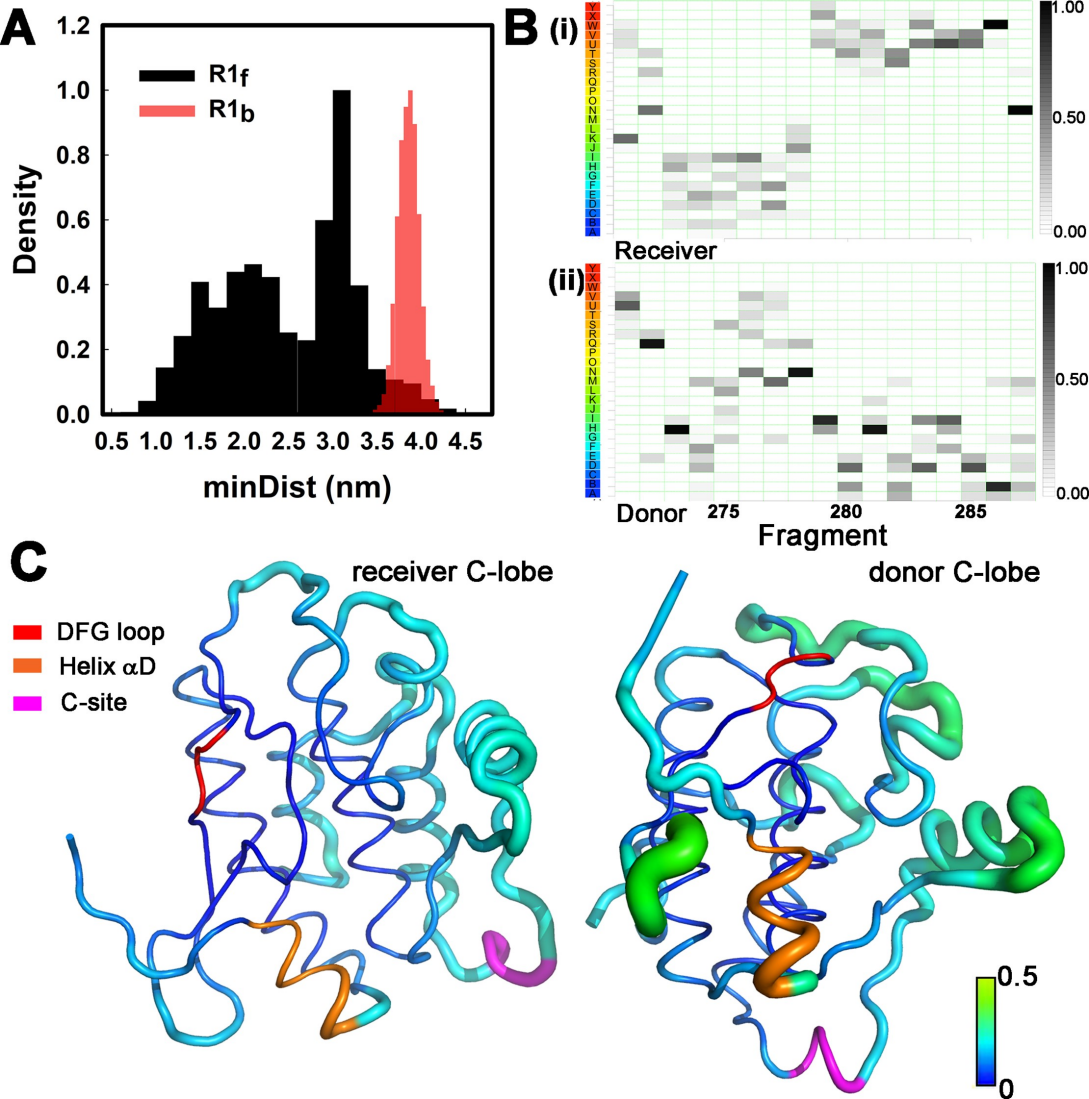
Capture strains R1 and freezes the receiver C-lobe. **A.** End-to-end distance distributions of the free (black (R1f) and bound (red (R1b)) R1 segments. **B**. SA distribution profile showing loss of α-helical character upon transition from (**i**) free to (**ii**) bound. The SA letters are color coded (red = α -helical -> blue = β sheet). **C.** Averaged RMSF fluctuations from combined MD replica runs (TPO form) mapped onto the 3KK8 dimer. R1 capture freezes out helix αD L_97_FEDIVAR_104_ (orange) and interface motions in the C-lobe of the receiver core. D_156_FG_158_ motif (red), C-site P_235_EWD_238_ (magenta). The vertical bar indicates RMSF value on the same colour scale shown in Fig 1B.

The normalized mutual information (*nMI*) provides an information theoretic measure of the coupling. This is consistent to the Shannon entropy *H*(*X*) for pairs of fragments albeit incomplete because of finite sampling [43]. We first compared the end-to-end distance distributions of the captured relative to the free R1 to assess constraint (**Fig 6A, S3 Movie, S4 Movie**). The captured R1 was constrained to a highly-extended subset of conformations. Secondary structure analysis based on the SA (**Fig 6B**) revealed the extended configuration resulted from melting of two donor R1 α-helical segments N_272_RERV and D_284_VDCL; while the intervening alanine-rich V_277_ASAI sequence largely retained α-helical character. The result was in line with mean residue helical propensity [44] of the fragments (-0.18 (V_555_ASAI) > -0.29 (N_551_RERV) and 0.49 (D_563_VDCL) kcal / mol). Our results are in excellent agreement with ESR experiments where spin labels were introduced at single residue positions throughout R1 in an inactivated monomeric *C*. *elegans* KD. The labels reported correlation times consistent with loop and α-helical configurations for residues S_278_AIHRQ and D_284_VDCL respectively [13].

We next focussed on the C-lobe to evaluate the compensatory decrease in flexibility of the core with greater precision (**Fig 6C**). Comparison of the RMSF profile of the dimer C-lobes shows that reduced flexibility of the B site helix αD and the interfacial surface of the receiver lobe are responsible for the overall decrease reported by PCA (Fig 5B).

### Electrostatics of T286 phosphorylation

The solvent exposed area and electrostatic properties of the phosphorylation site were calculated for the most populated conformations extracted by cluster analysis. Dimer conformations from each set of MD replicas, as well in the tCONCOORD ensemble with the phosphomimic mutation (TPO286D) [1] were analysed. Dimer contacts were localized to the C-lobes. Contact between R1 of the donor subunit with the receiver subunit core upon capture accounted for much of the stabilization due to solvation. The total buried surface area due to subunit capture was 2319±42 A^2^. The contribution to this value due to R1 capture was 1765±81 A^2^ (~ 75%). The free-energy difference due to the decrease from solvation was twice as great for the phosphorylated (-7.4 kcal/mole) versus the non-phosphorylated (-4.2 kcal/mole) form.

**Fig 7:**
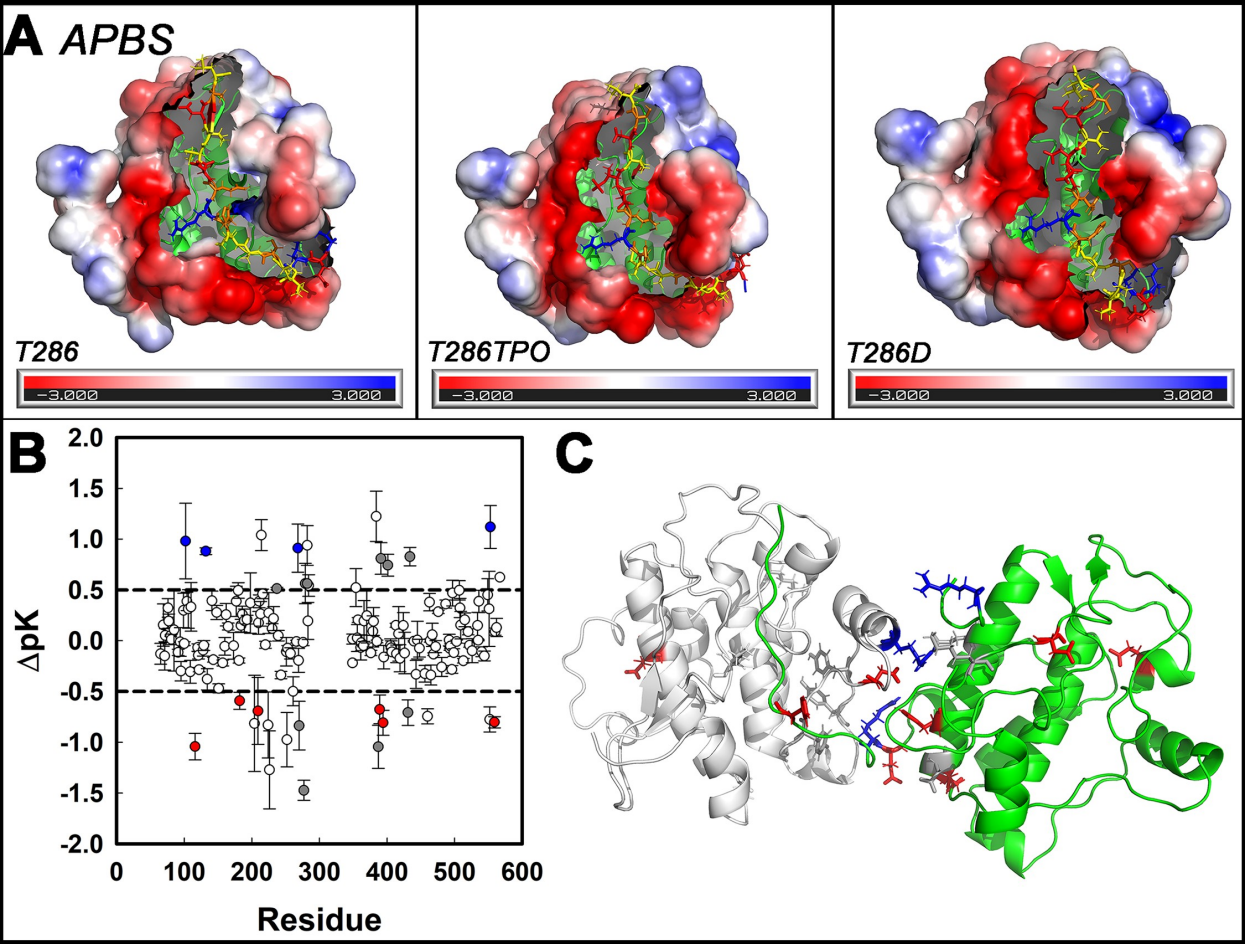
Primary site phosphorylation increases R1 cross-talk. **A.** Changes in the electrostatic environment (red = negative (acidic), blue = positive (basic), white = neutral (non-polar) triggered by T286 modification (-> TPO phosphorylation or -> D mutation). The binding crevice becomes more negative while the adjacent subunit interface becomes more positive. Color scale is -3 to + 3 kT/e mV. **B.** Phosphorylation dependent residue pK shifts (mean ± standard error) of polar residues (n = 73). Colors denote residues shifted towards negative (red), positive (blue) or more neutral (dark grey) values. **C.** Polar residue (stick side-chains; colors as in B (n = 146)) pK shifts > 0.5 mapped onto the C-lobes of the 3KK8D.pdb dimer. The computed net pK change upon phosphorylation is +1.95 ΔpK / dimer (0.0135 ± 0.005 ΔpK (0.8 ± 0.3 kBTe mV) / residue).

Computed Poisson-Boltzmann electrostatic fields are shown in (**Fig 7A**). The R1 binding pocket was more acidic and the dimer interface more polar when T286 was phosphorylated (TPO286) or mutated (D286). Polar residues with large (> 0.5) pK shifts between the T286 and TPO286 forms were identified by comparison of the dominant cluster configurations (**Fig 7B**). These residues localized to the C-lobes around the R1 binding pocket and the subunit interface (**Fig 7C**). Phosphorylation shifted the R1 binding pocket surface charge to negative values, while its interior became more polar. The measured pK shifts accounted for the more rigid interface.

### The allosteric network forms a self-reinforcing R1 relay

**Fig 8:**
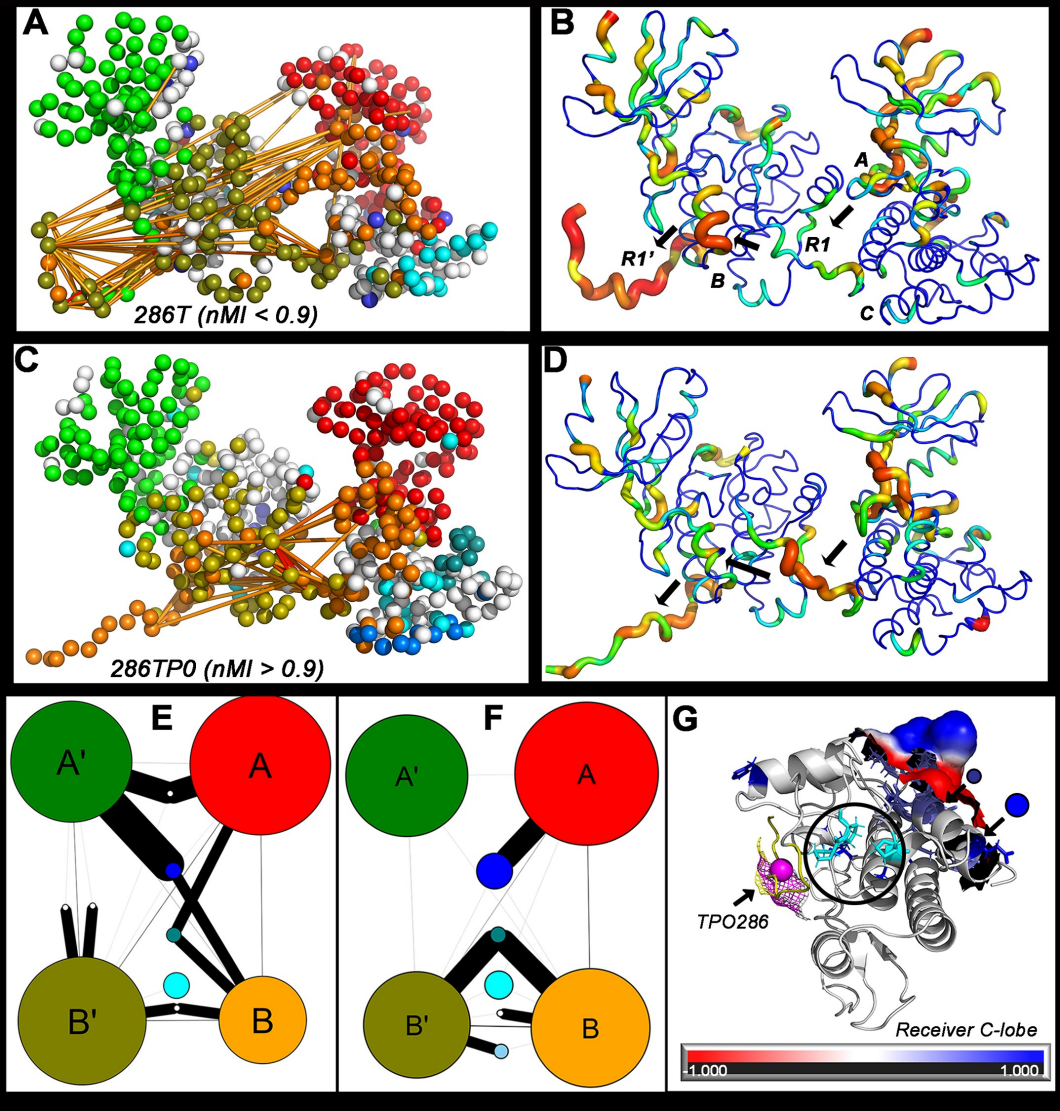
Relays and communities mediating R1-R1 signal processing. **A.** Community membership and top couplings (yellow lines) of the native dimer. **B.** Eigen-centrality map of the native dimer. **C.** Community membership and top couplings (orange lines) of the phosphorylated (TPO) dimer. **D.** Eigen-centrality map of the TPO dimer. G -> R color code (bar) and thin -> fat backbones indicate increasing values. Arrows (black) indicate the shortest pathway linking the substrate subunit A site and R1 with the enzyme subunit B site and R1. **E, F.** Community graphs for the native (E) and TPO (F) dimer show parallel signal processing between the donor R1 (in community B) and the receiver R1 (in community B’). **G.** Surface protrusion and electrostatics (-1 (red) to +1 (blue)) of the receiver KD protein sector (249D-259R, 263D) that forms the intermediate community (teal sidechains) mediating the dominant B -> B’ signal relay in the TPO286 dimer. The more distributed signal community mediating the A -> B’ signal relay (blue sidechains) increases size by recruiting the same sector from the donor KD upon phosphorylation. Three proline residues (P212, P213, P235) (cyan sidechains) targeted by human mutations (circled) cluster between the captured TPO286 (magenta sphere) and the teal community. R1 (yellow).

Community membership mapped onto structure revealed the captured R1 segment and its binding pocket formed a central community (B’) distinct from A’. B’ had strong interactions with donor KD communities A and B. The adjacent R1 segments were linked by long-range couplings between B’ and B. The nucleotide binding pocket and adjacent fragments of its donor KD also linked to the captured R1 (**Fig 8A**). Superposition of the network centrality profile onto the structure delineated a signal relay from the donor A-site to the captured R1 via helix αD (B-site) in the receiver to its R1 (**Fig 8B, S5 Movie**).

R1-R1 communication was increased upon phosphorylation (T -> TPO) due to the creation of strong couplings (nMI > 0.09) between captured R1, the donor A and receiver B and C sites (**Fig 8C**). These couplings represented the tail of the nMI distribution between the 2σ -3σ significance level (**S4 Fig**). The dominant signal relay was remarkably similar to that formed by the top couplings from the tCONCOORD TPO286D ensemble (Fig 3). Superposition of the TPO dimer centrality profile highlighted the relay was localized to the captured R1, donor A, C and receiver B sites (**Fig 8D**).

Parallel network signal processing in the networks was quantified with community size (number of residues) and interaction strength represented, as in Fig 2B, by community graphs (**Fig 8E and 8F**). Phosphorylation strengthens B -> B’ interactions at the expense of A’ interactions. Two small communities mediated A -> B’ and B -> B’ signal relays. These were generated upon R1 capture and strengthened upon phosphorylation. Most human mutations causing learning disabilities map within or close to sectors that form part of the R1-R1 relay network. A sector common to both communities comprised a loop with a proline cluster (P212, P213, P235) between C-lobe α-helices 9 and 10 that is well conserved, polar in character and at the opposite face to the R1 binding site (**Fig 8G, S6 Movie**). The P212 mutation has an abnormal electrophysiological phenotype [18], though it does not influence binding interactions. The loop is an attractive target for the design of positive allosteric modulators based on these characteristics. The proline mutations could also regulate interface flexibility in addition to communication between RI and the intermediate relay communities (Fig 7G),

### Allosteric coupling and kinase cooperativity

**Fig 9:**
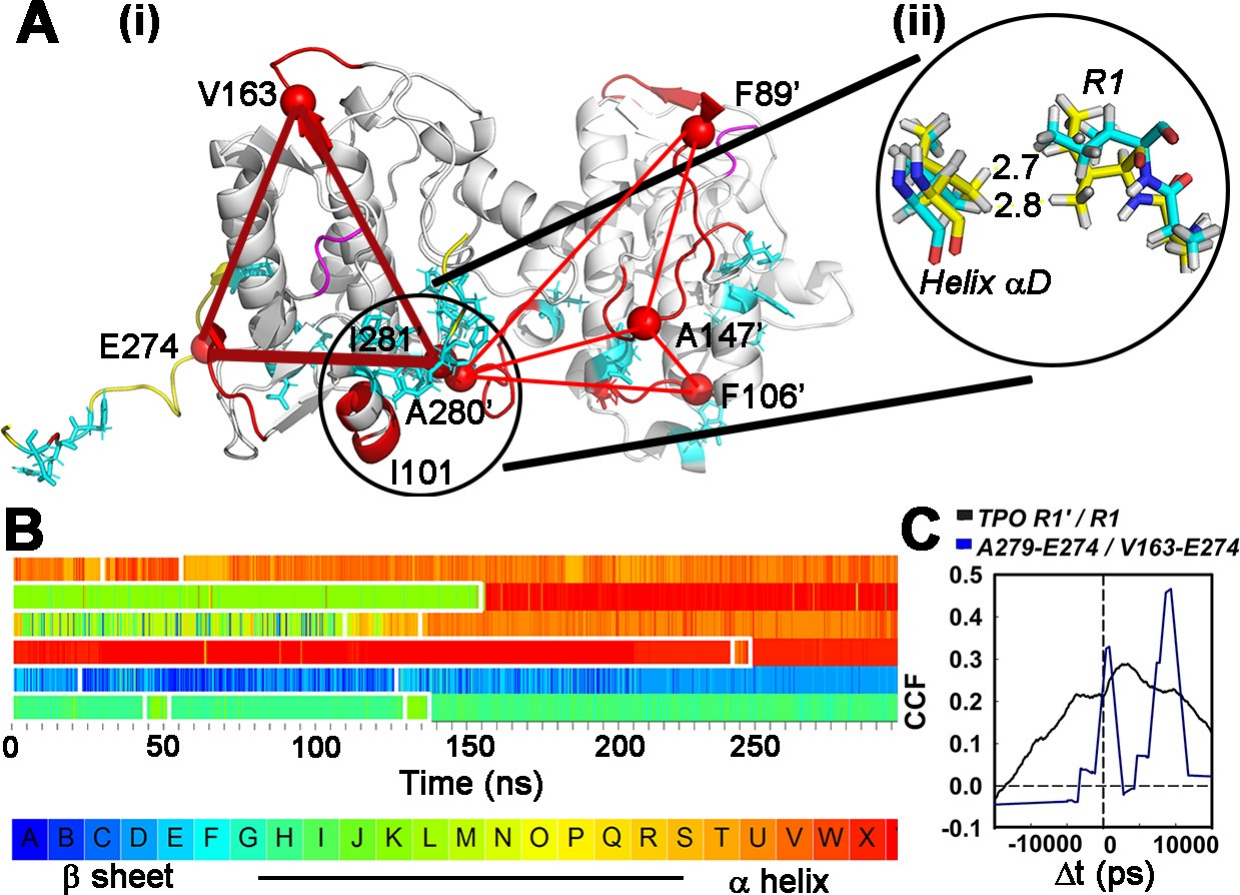
Temporal couplings of the R1-R1 signal network. **A. (i)** Parallel signal pathways formed by the dominant, long-range couplings in the C-lobe. The hydrophobic patch (circled) formed by helix aD and captured R1 orchestrates the couplings. Two pathways (1 direct (thick red line), 1 indirect (thin red lines) connect the patch and the free R1. The donor C-lobe also links to the patch (dotted red lines). R1 segments (yellow) and DFG motifs (purple) are highlighted. Cyan side-chains show residue positions where mutations cause learning disabilities in humans. Fragment labels (type, residue number) **(ii)** atomic (stick) representation of the hydrophobic patch in the major configurations derived from cluster analysis of the T (cyan) and TPO (yellow) forms. **B.** Times series from one replica run of structural transitions within the signal pathway fragments (Top -> Bottom: I101,V163,E274.A147',A280',A281'). Colours denote SA letters as in 6B. **C.** CCF for couplings between R1 segments in the phosphorylated (TPO) dimer end-to-end distance fluctuations (black line) and couplings for direct and indirect pathways (blue line).

Transmission of information between subunits encoded by the phosphorylation-dependent couplings was analysed to understand signal relay kinetics. The core network of long-range allosteric couplings (n = 115, nMI > 0.09) linked the bound and free R1 to sites A, B and C (**Fig 9A**). A patch of three hydrophobic residues that attach R1 to B site helix αD (I101, A280”, I281”) formed the central node. This node is less compact in the native (T286) relative to the phosphorylated (TPO286) dimer.

*C*.*elegans* CaMKII mutations I101D and I281D in the hydrophobic patch that replace the non-polar isoleucines with charged aspartates abolish cooperativity (Hill coefficient 1.4 -> 1.0) in decoy dimers [8] in agreement with our simulations. Similar measurements are not available for either TPO286D and TPO286A, but extensive measurements of multiple parameters in vitro and cell culture have established that these mutations mimic the phosphorylated and dephosphorylated forms of the enzyme respectively [1]. We generated tCONCOORD ensembles of engineered mutations *in silico* to compare the eigenvector centrality profile of a double I101D.I281D mutant with those of TPO286D or TPO286A. Comparison of the profiles implied a better match between I101D.I281D and TPO286A, with differences from TPO286D localized to two segments (**S5 Fig**). This suggests that the TPO286A phenotype may result from loss of cooperativity; while the TPO286D phenotype has increased cooperativity.

### Temporal correlations within the R1-R1 relay

Time series of the local, SA-encoded structural transitions for residue fragments that constitute this phosphorylation-dependent network are plotted for one replica run (**Fig 9B**). The most frequent transitions were short (< 10 ns) and sampled a restricted range of dihedral angles. Dihedral angle jumps scale with the separation between SA letters. Large jumps were rare but persisted for longer times (> 100 ns) when they occurred. Two examples are shown. The large jump for fragment V163 loop to α-helix is around the middle (~150 ns) of the time series. There is a smaller jump for fragment E274 between loop (β-sheet to α-helix) configurations at a similar time.

The cross-correlation (CCF) between the nMI time-series for the long-range (> 12 angstrom) couplings in the direct (A279-E274) and indirect (V163-E274) R1-R1 pathways in the phosphorylated (TPO286) dimer network showed two peaks consistent with the short duration of fragment structural transitions (1–2 ns τ_1/2_ ‘s) (**Fig 9C**). The first peak (Δt < 1 ns) presumably represents couplings associated with the direct pathway. The second peak (Δt ~ 10 ns) represents the lag associated with the first stage of the indirect, two-stage pathway. The CCF for the end-to-end fluctuations of the two R1 segments in the phosphorylated dimer reported a weak correlation (amplitude = 0.35, correlation time τ = 15 ns). The correlation was not significant for the non-phosphorylated dimer.

## Discussion

Our findings indicate that the captured R1 freezes collective motions of the receiver subunit C-lobe to orchestrate allosteric coupling based on helix melting. Secondary structure fluctuations revealed α-helical character of free R1 compatible with ESR evidence [13] and showed that the captured RI transitions to an extended conformation energized by binding site solvation. The bound R1 triggered long-range couplings that propagated to the receiver R1. R1 is similar to α-helical linkers in the bacterial flagellar basal body that also respond to strain [45]. Such “chameleon” helices may play essential roles in propagating conformational states across multiple subunits. While initial R1 capture has an enthalpic contribution, subsequent signal propagation is entropic as found for many systems [46].

The allosteric network **(1)** has a global architecture based upon a generic monomer KD network; **(2**) has retained nucleotide and substrate binding site couplings important for activation; **(3)** is strengthened by T286 phosphorylation and **(4)** incorporates mutated residue positions implicated in human disabilities as well as possible therapeutic targets. We discuss these network properties below.

### Global architecture

Dynamic couplings consider both population shifts and propagation timescales of conformational ensembles [47]. Thus, they provide a more refined readout for mechanistic analysis than the architecture of the protein fold [48]. The auto-inhibited CaMKII form is completely inactive since the ATP binding site is not in an optimal conformation and the substrate binding site is occluded by the regulatory segment. The multiple community membership of the DFG and helix αD sites drove global adaptations of the network architecture coupling nucleotide and substrate binding. Reduced coupling between the communities, most marked for the coiled-coil dimer (BDW) structure, characterized the inactivated state. R1 capture results in reduced flexibility of the receiver C-lobe core; reported by RMSF, PCA, cluster and community network analysis.

The monomer network is co-opted in the chained dimer to generate a network that spans both KDs. A hydrophobic patch between the R1 central residues and helix αD orchestrated R1_donor_-> R1_receiver_ signal relays; most dominantly from the patch directly to R1_receiver_ N-terminal segment and indirectly via the receiver DFG motif. Interfacial interactions linked the donor DFG motif to the patch. The prominent fragment couplings lasted a few to a hundred nanoseconds. Most were confined to short (~ 10 ns) transitions over a small conformational range. Large conformational transitions were infrequent but persisted for longer (>100 ns) times. Transitions between phosphorylated and dephosphorylated conformations completed within a hundred nanoseconds. The temporal behaviour is consistent with a complex free-energy landscape with multiple pathways [49]. We suggest the reduced flexibility of the receiver KD C-lobe upon R1 capture will influence the displaced receiver R1 to explore and bind to adjacent “open” subunits with floppy cores rather than re-associate with its own frozen, occupied core. The outcome is a self-reinforcing network that can be serially transmitted across the ring of KD C-lobes.

### Activation dynamics

Our survey of CaMKII KD monomer structures revealed the nucleotide binding DFG motif and the B-site αD helix as conserved, coupled network nodes. The DFG motif is OUT when the KD is bound to the AD hub; an interaction analogous to the docking interaction of the PKA catalytic subunit with its regulatory subunit [7]. There were two distinct DFG IN orientations, one associated with activated and the other with inactivated states, as in Aurora kinase (AurA) [50]. The DFG motif is IN in auto-inhibited KD’s not bound to the hub. Thus, DFG motif orientation may report on compact and extended inactive holoenzyme states that have been visualized by EM cryo-tomography [51]. In contrast, helix αD is OUT in all auto-inhibited, but IN for activated open and substrate-bound structures.

Our results add to data arguing that while the DFG motif is an important determinant of ATP binding its mobilization follows different strategies during activation of EPKs. In AurA, activation by the effector TpX2 involves an equilibrium population shift from the OUT to IN state [52]; but activation by phosphorylation triggers a switch between auto-inhibited and activated IN states [50]. Multiple strategies for kinase auto-inhibition have also been identified, for example, in the ZAP-70 tyrosine kinase [53].

Cooperativity in BCR-ABL, a tyrosine kinase identified as a hallmark for myeloid leukaemia can be either negative [54, 55] or positive [26] depending on the coupling between the nucleotide and substrate binding sites. In CaMKII, as reported here, the nucleotide and substrate binding modules are again recruited for the formation of positive trans-subunit couplings. Comparative bioinformatics reveals common substrate binding site interactions between CaMKII and phosphorylase kinase [56], but the phosphorylase holoenzyme is constitutively active and its complexity limits the study of nucleotide-substrate coupling.

### The link between behaviour and protein dynamics

The histological, cellular and biochemical phenotypes of the human mutations that cause intellectual disabilities have been documented [18, 19]. Brain imaging and electrophysiological recordings revealed cerebellar atrophy and abnormal action potentials (APs) respectively in many mutants. The common biochemical correlate was altered phosphorylation levels of the primary phosphorylation site (T286).

T286 trans-phosphorylation has multiple short and long-term consequences for LTP. In the short-term second time scale, the optimal electrical stimulation frequency for LTP in neurons triggers dissociation from the actin cytoskeleton for sequestration at the PSD in dendritic spines. Synaptic localization of CaMKII holoenzymes by the triggered millisecond Ca^2+^ transients [57] persists over an hour [15], implying the activated CaMKII transitions to a long-lived state. *In vitro* experiments report the Ca^2+^/CaM affinity for CaMKII R2 increases more than 1000-fold; essential for the frequency-dependent activation [29, 58]. The activation triggers sub-second dissociation from actin and subunit exchange over minutes to propagate the activated state to previously inactive holoenzymes and prolong the lifetime of an activated holoenzyme population [59]. The phosphorylation also alters the affinity of some substrates for CaMKII, thus modifying substrate selection [60, 61]. These multiple effects are all likely to arise from allosteric disruption of kinase cooperativity that would alter the optimal frequency [62] at which the holoenzyme responds to AP-triggered pulsatile calcium stimuli. Our MD simulations develop an initial allosteric framework responsible for the kinase cooperativity.

Simulations of crystal structures of activated dimer complexes resolved changes triggered by dimerization and T286 phosphorylation. The match of the dimer RMSF to crystal B-factors indicated differential flexibility of the linked kinase domains. We found that conformational perturbation of the captured R1 helix drove the flexibility change to orchestrate the allosteric network. The R1 conformational perturbations reported by our simulations agree well with ESR analysis of R1 fragments in KDs [13]. The ESR study used an inactive, docked versus active, captured R1 two-state model to interpret the data. The free R1 would also form a third open state, as recognized in our comparative analysis (Fig 6A), with similar ESR signature to the docked state; but the study did not examine the concentration dependence that could have distinguished between these states. The greater stability of the phosphorylated dimer is also consistent with ESR evidence that captured R1 lifetimes are increased in the phospho-mimic mutant T286E.

T286 phosphorylation strengthened the coupling between chained KDs, with pK shifts of buried residues towards neutral values and surface, interfacial residues towards polar values, accompanied by compaction of the hydrophobic patch. The nanosecond coupling dynamics, relevant for the accommodation of the rapid changes in calmodulin and actin affinity, could be low-pass filtered in the dodecameric holoenzyme to freeze the activated cores over millisecond times after Ca^2+^/CaM dissociation to allow for diffusion of activated holoenzymes to the PSD. Similarly, during subunit exchange, the activated dimer must outlast the time for encounter with another holoenzyme. Diffusion and encounter times are on the order of milliseconds based on known spine volumes (~ 1 μm^3^) and spine CaMKII concentrations (> 0.1 M). Conformational spread powered by strong inter-subunit coupling can achieve long-lived configurational states over seconds in multi-subunit ring assemblies such as the bacterial flagellar motor [63].

Structural elements that regulate the positive CaMKII kinase cooperativity have been identified. Reported Hill-coefficients (*H)* for substrate phosphorylation with Ca^2+^/CaM concentration varied from 4.3 to 1.1 in the *C*. *elegans* holoenzyme accompanied by substrate-dependent shifts in half-maximal dose. The *H* value is, for instance, downshifted by inhibitor peptides (*H* 4.3 -> 1.7). Importantly mutations (F98E, I101D, I205K) that introduce charged residues within or adjacent to the hydrophobic patch reduce dimer cooperativity in native / decoy KD mixtures (*H* 1.5 -> 0.9); while the I -> D mutation in R1 residue 281D reduced holoenzyme cooperativity (*H* 3.0 -> 1.5) [8]. Thus, available kinase cooperativity data agree qualitatively with the main features of the allosteric network we have characterized.

A screen of tCONCOORD ensembles revealed the changes in the dimer allosteric network caused by in silico mutations TPO286A and I101D-I281D are similar and distinct from TPO286D (S5 Fig). The change in kinase cooperativity with T286 phosphorylation, or *H* values for the phosphorylation (TPO286D) and dephosphorylation (TPO286A) mutant mimics, have not been reported. A triple alanine mutant (T286-305-306A) does not seem to affect cooperativity [8] but interpretation requires further work since phosphorylation of the inhibitory T305-T306 sites is antagonistic to T286 phosphorylation [1].

The relationship between inter-subunit coupling and cooperativity has been extensively modelled and experimentally characterized in the bacterial flagellar motor, a single ring assembly. Similar elucidation for CaMKII will be more challenging for this two ring dodecamer. Its architecture raises multiple possibilities for trans-phosphorylation [62]. Two distinct modes for conformational coupling have been proposed; a lateral spread of the activated conformation across the holoenzyme subunits [8] or transverse paired dimers [9, 32] (**S6 Fig**). The latter may also mediate activation-triggered subunit exchange via the central AD hub [64]. Interestingly. consistent with this idea, kinase cooperativity varies inversely with inter-domain linker length (*H* 4.3 –> 1.7) [8] in the *C*. *elegans* holoenzyme and is reduced (*H* 2.1 -> 1.1) by a mutation in the AD hub interface docking the KD DFG motif in the human holoenzyme [7]. The present study is an important first step towards this elucidation. It provides the first detailed framework of the allosteric network to reveal self-reinforcing R1 signal relays facilitate inter-subunit couplings that underlie any cooperativity mechanism.

### Pharmacological implications

The mutated residue positions responsible for impaired human behaviour are distributed along the allosteric signal relays generated by R1 capture and strengthened by T286 phosphorylation. Thus, the probable molecular rationale for these mutations is that they principally act to disrupt the allosteric network rather than weaken substrate or nucleotide binding per se. Disruption of the allosteric network could have multiple outcomes as enumerated above to produce gain or loss of function with diverse pathophysiological consequences. Importantly, this study opens possible avenues for therapeutic treatment. MD simulations have proved useful for determination of the efficacy of peptide inhibitors to ATP-binding pocket residue mutations [65]. The design of allosteric inhibitors is more challenging as it requires an evaluation of the conformational plasticity of the protein assembly but promises greater specificity. We have identified protein sectors based on community analysis that may disrupt conformational coupling without altering calmodulin, nucleotide or substrate binding. Their predicted role as signal relays rather than binding determinants can be tested by mutant screens. An excellent example in the literature of such a mutation is PKA Y204A that disrupts coupling between nucleotide and substrate binding without affecting binding affinities [66].

In conclusion, our simulations make the case that the conformational dynamics of chained dimers have advantageous properties for subunit exchange and holoenzyme activation. Network analysis revealed the centrality of the coupled A (DFG motif) and B (helix αD) sites in the R1 relay to suggest how substrate affinity is modulated by nucleotide occupancy and how both influence cooperativity. It detailed how T286 phosphorylation strengthened conformational coupling initiated by R1 capture. Finally, community graphs identified targets for the rational design of allosteric inhibitors [22]. Future work will build on these advances to reconcile the dynamic architecture of the kinase with measurements of its cooperativity.

## Materials and methods

### Phylogenetics

CaMKII sequences were retrieved from Uniprot [67]. MUSCLE was used for multiple sequence alignment (MSA). Secondary structure predictions were made with PsiPred. The MSA was manually curated in Jalview. The phylogenetic tree was constructed with Fast-Tree 2.19 and displayed with Fig-Tree 4.3 (*http*:*//tree*.*bio*.*ed*.*ac*.*uk/software/figtree/*).

### Structure Preparation

The human δKD structure alone (PDB ID: 2VN9) and with calmodulin (2WEL)[9], complete subunit from the human holoenzyme (3SOA [7]); the *C*. *elegans* KD structures alone (2BDW [23], 3KK8 [8]) and with CaMKIINtide (3KL8 [8]) and the open form of protein kinase A (1CMK [68]) were downloaded from Protein Data Bank. Missing atoms were added in Swiss-PDB viewer (*www*.*expasy*.*org/spdbv*); missing loop segments with Modeller (*https*:*/salilab*.*org/modeller*). Mutant substitutions were made in Pymol (*http://pymol.org*) then energy minimized in Modeller.

### tCONCOORD

Parameters for tCONCOORD runs were as detailed earlier [69]. tCONCOORD utilizes a set of distance constraints, based on the statistics of residue interactions in a crystal structure library [25, 70], to generate conformational ensembles from an initial structure without the inclusion of solvent. Sets of 16^4^ = 65,536 equilibrium conformations with full atom detail were typically generated for each structure. The overlap between ensemble subsets was > 99% when the subset size was < 1/4 of this value [69].

### Molecular dynamics

A set of 3 replicas of 300ns were generated for *E*. *coli* 3KK8 structure and its 286T equivalent using GROMACS 2016.2 with Amber ff99sb*-ILDNP force-field [71]. Each system was first solvated in an octahedral box with TIP3P water molecules with a minimal distance between protein and box boundaries of 12 Å. The box was then neutralized with Na^+^ ions. Solvation and ion addition were performed with the GROMACS preparation tools. A multistage equilibration protocol, modified from [72] as described in [33], was applied to all simulations to remove unfavourable contacts and provide a reliable starting point for the production runs including: steepest descent and conjugate gradient energy minimisation with positional restraints (2000 kJ mol^-1^ nM^-2^) on protein atoms followed by a series of NVT MD simulations to progressively heat up the system to 300 K and remove the positional restraints with a finally NPT simulation for 250 ps with restraints lowered to 250 kJ mol^-1^ nM^-2^. All the restraints were removed for the production runs at 300 K. In the NVT simulations temperature was controlled by the Berendsen thermostat with coupling constant of 0.2 ps, while in the NPT simulations the V-rescale thermostat [73] was used with coupling constant of 0.1 ps and pressure was set to 1 bar with the Parrinello-Rahman barostat and coupling constant of 2 ps [74]. A set of 3 replicas with time step 2.0 fs with constraints on all the bonds were used. The particle mesh Ewald method was used to treat the long-range electrostatic interactions with the cut-off distances set at 12 Å. The threonine phosphate (TPO286) was changed to threonine (T286) after equilibration to generate the non-phosphorylated form. The 300 ns MD runs reached stationary root mean square deviation (RMSD) values within 3 ns.

### Energetics.

Contact residues, surfaces and energies were extracted from the PDB files with the sub-routines (*ncont*, *pisa*) available in CCP4 version 7 (*http*:*//www*.*ccp4*.*ac*.*uk/*). Continuum electrostatics were computed with the Poisson Boltzmann solver (*http*:*//www*.*poissonboltzmann*.*org/*) [75]. Comparison with experimental B-factors and geometrical analyses were performed with GROMACS version 4.5.7 (*http*:*//www*.*gromacs*.*org/*).

### Structural alphabet

The mutual information *I*(*X*;*Y*) between two variables (*X*) and (*Y*) is

I(X;Y)=H(X)+H(Y)−H(X,Y);

where *H*(*X*,*Y*) is the joint probability distribution;

The normalized mutual information, *nMI*(*X*;*Y*) = (*I*(*X*;*Y*)−*ε*(*X*;*Y*))/(*H*(*X*,*Y*));

*H*(*X*) is a measure of the entropy Δ*S*(*X*) that is related to the number of microstates and their probability. *k*_*B*_ Is the Boltzmann constant

$$\begin{array}{c} \Delta S(X)=k_{B.}\ln\left(W_{X}\right)=k_{B}.\sum_{i=1}^{n}{p(X}_{i}).\ln\left(p(X_{i})\right)\\ H(X)=\Delta S(X)/k_{B.} \end{array}$$

*ε*(*X*;*Y*) is the expected, finite-size error. The finite-size error estimated as in earlier publications (e.g. [45, 76]) corrects for the effects of finite data and quantization on the probability distribution [77].

The *nMI* couplings are detected as correlated changes in fragment dynamics, after spatial filtration to isolate long-range couplings [76]. In methods based on the dynamics cross-correlation matrix [78], the correlation is calculated from position fluctuations of the residue C^α^ atoms. In the MutInf method [79], correlations are measured in terms of mutual Information between discretized torsional angles. The residue-based approaches do not directly read-out couplings between structural motifs, e.g. secondary structures. The SA [80], is a set of recurring four residue fragments encoding structural motifs derived from PDB structures. There is no need for discretisation and / or optimisation of parameters as the fragment set is pre-calculated.

### Principal component analysis

Collective motions were identified by PCA of the conformational ensembles. PCs were generated by diagonalization of the covariance matrix of C^α^ positions in GROMACS 4.5.7. The overlap (cumulative root mean square inner product) of the PCs between replicas [40]) and the PC dot product matrix was computed with the GROMACS g-anaeig function. The motions have no time-scale for tCONCOORD ensembles, but comparison with MD trajectories was consistent with the notion that collective motions represented by the first few PCs are “slow” relative to smaller amplitude motions recorded by the later PCs.

### Network analysis

Statistically significant correlations between columns were identified with GSATools [36] and recorded as a correlation matrix. The correlation matrix was used to generate a network model; with the residues as nodes and the correlations as edges. The contribution of a node to the network was estimated by the eigenvector centrality, *E*, calculated directly from the correlation matrix:

$$E.{\left[M\right]}_{corr}=E.\lambda$$

where [*M*]_*corr*_ is the correlation matrix and λ is the eigenvalue

The Girvan–Newman algorithm [37] was used to identify community structure. Then the network was collapsed into a simplified graph with one node per community, where the node size is proportional to the number of residues. Edge weights represent the number of nMI couplings between communities [38]. Community analysis of correlation networks identifies relatively independent communities that behave as semi-rigid bodies. Graphs were constructed with the *igraph* library [81] in R (*https*:*//cran*.*r-project*.*org/web/packages/igraph/*) and visualized in Cytoscape (*http*:*//www*.*cytoscape*.*org/**)*.

### Quantification and statistical analysis

The typical size of a tCONCOORD ensemble was 65,536 conformations (256^2^). Three MD replicate runs for the two (phosphorylated, dephosphorylated) conformations. Ensemble conformations and MD runs were averaged for computation of the nMI between fragment positions, with > 2σ threshold for selected top couplings. Pearson’s correlations were used for comparison. Significance limits were set in GSATools. **S1 Table** lists the web databases and software servers used in this study.

## Supporting information

S1 Fig CaMKII KD Conservation. **A.** Multiple sequence alignment (MSA) of human (green), rat (blue) and the nematode C. elegans (red) isoform and splice variant sequences. The crystal structure library contains structures from nematode and human species. The rat enzyme has been extensively characterized by mutagenesis, biochemical and behavioural assays. Residue colour (JalView–Zappo) denotes type. Predicted secondary structure (β sheet (yellow bars); α helix (magenta bars)). **B.** Tree constructed from CaMKII sequences in the Uniprot database (>500) shows the phylogenetic distance of the vertebrate rat/human (blue/green) sequences from the invertebrate (red) nematode. **C.** B-factors (black) from the 3KK8 crystal structure compared against simulated factors from tCONCOORD (green) and MD (blue). Secondary structure (β-sheet (yellow); α-helix (red)). Crystal contacts (brown). **(i)** Donor KD (**Inset**: Cartoon representation showing secondary structure (Mg^2+^ (magenta sphere))) **(ii)** Receiver KD.
(PDF)

S2 Fig PKA and CaMKII have Homologous Structure and Dynamics. **A.** Superposition of the *C*. *elegans* CaMKII KD (3KK8 [8]) with the open (1CMK [68]) form of PKA, a paradigm for analysis of EPKs, emphasize the canonical kinase fold. The structural homology is accompanied by sequence homology (E value < 10^−5^); but different quaternary structure. PKA is a tetramer with distinct catalytic and regulatory subunits, rather than a dodecamer with catalytic and regulatory domains in a single subunit as in CaMKII. PKA has an IQ motif for calmodulin binding and a flexible N-terminal helix upstream of this motif. The fit RMSD = 0.48 angstroms based on BLASTP multiple sequence alignment of 1CMK residues 41–312 with 3KK8 residues 11–286. **B.** PKA (1CMK) in the orientation shown in [26]. The community map obtained with tCONCOORD was consistent with, albeit coarser, than the published map derived from MD simulations [26] with four major communities of matching size and location. Smaller communities were not resolved probably because tCONCOORD does not account for anisotropic residue motions and solvent is implicit. **Box:** Community color coding as in [26].
(PDF)

S3 Fig Eigenvector over-lap. **A**. Cumulative overlap (root mean square inner product) [42] between pairs of the three 3KK8d_TPO replicas (mean (± se). **B.** Mean values for the 9 vector dot products between PCs 1–3 averaged over the 3 replicas.
(PDF)

S4 Fig Selection of top couplings from the nMI distribution. The tail (red) of the total (white) nMI distribution in the 2σ– 3σ range was analyzed for the top nMI couplings. Within this range formation of the R1-R1 relay dictated the choice of threshold (nMI = 0.09 (2.2 σ) for 3KK8d_TPO distribution shown). Snapshots at different thresholds (0.12 -> 0.10 -> 0.08) illustrate how the network forms starting with the initial central node. The selected nMI threshold for the 3KK8d-T distribution at similar significance level (2.2 σ) was 0.06.
(PDF)

S5 Fig tCONCOORD network centrality of inactive mutant dimers. Comparison against 3 ensembles of the phospho-mimic TPO286D (open symbols, black line; error bars (se)). Ensemble size = (128^2). **(i)** TPO286A (blue). **(ii)** I101D.I281D. Circles represent S, T and phosphorylation sites as in Fig 2A. Asterisks indicate upshifts (red) and downshifts (black) in the phospho-mimic relative to the mutants. Lines denote means ± σ (dashed ± dotted). **(iii)** Running filtration of 20-fragments isolates two sequence segments (fragments 41–125; fragments 375–415) whose centrality is strengthened by activation (red bars). Pearson correlations (TPO286D–TPO286A; TPO286D-I101D.I281D; TPO286A-I101D.I281D) are 0.86, 0.89, 0.95 (Total), 0.55, 0.73, 0.88 (41–125) and -0.2, 0.34, 0.68 (375–415) respectively.
(PDF)

S6 Fig Conformational coupling in the CaMKII holoenzyme. KDs in upper (light grey) and lower (dark grey) rings hexamer rings arrayed the central hub (white circle). Dashes (red) indicate couplings (number = n). **A**. Paired dimers form but do not interact. In absence of other interactions, H < 2. **B**. Subunits in each ring are conformationally coupled. H values > 2, and R1 dependent formation of KD multimers in solution [7, 9] are more straightforwardly reconciled with lateral conformational spread.
(PDF)

S1 Table Web resources–Software and databases. (PDF)

S1 Movie PCA of the 3KK8 monomer (AVI). The three major communities are colour-coded as in Fig 2B. TPO286 (red spheres). The filtered PC, PC1 and PC2 motions are shown. Related to Fig 5.
(AVI)

S2 Movie PCA of the 3KK8 dimer (AVI). The three major communities are colour-coded as in Fig 2B. TPO286 (red spheres). The filtered PC, PC1 and PC2 motions are shown. Related to Fig 5.
(AVI)

S3 Movie Filtered PC trajectory of the free R1 (AVI). Free R1 motions from three MD replicas of the non-phosphorylated 3KK8 dimer sampled at 600 ps. T286 (yellow sphere). Residues are color-coded according to type (white = hydrophobic, red = acidic, blue = basic). Related to Fig 6A.
(AVI)

S4 Movie Filtered PC trajectory of the captured R1 (AVI). Captured R1 motions from three MD replicas of the non-phosphorylated 3KK8 dimer sampled at 600 ps. T286 (yellow sphere). Residues are color-coded according to type (white = hydrophobic, red = acidic, blue = basic). Related to Fig 6A.
(AVI)

S5 Movie Network architecture of the phosphorylated 3KK8 dimer (AVI). Related to Fig 8C.
(AVI)

S6 Movie Surface profile and spatial relationship of intermediate relay community residues with the proline cluster targeted by human mutations (AVI). Related to Fig 8G
(AVI)
