# Supplementary material for: Conformational coupling by trans-phosphorylation in calcium calmodulin dependent kinase II
Source: PLoS Comput Biol. 2019 May 31;15(5):e1006796. doi: 10.1371/journal.pcbi.1006796 (PMC6576796; doi:10.1371/journal.pcbi.1006796)
Supplement: S1 Fig — A. Multiple sequence alignment (MSA) of human (green), rat (blue) and the nematode C. elegans (red) isoform and splice variant sequences. The crystal structure library contains structures from nematode and human species. The rat enzyme has been extensively characterized by mutagenesis, biochemical and behavioural assays. Residue colour (JalView–Zappo) denotes type. Predicted secondary structure (β sheet (yellow bars); α helix (magenta bars)). B. Tree constructed from CaMKII sequences in the Uniprot database (>500) shows the phylogenetic distance of the vertebrate rat/human (blue/green) sequences from the invertebrate (red) nematode. C. B-factors (black) from the 3KK8 crystal structure compared against simulated factors from tCONCOORD (green) and MD (blue). Secondary structure (β-sheet (yellow); α-helix (red)). Crystal contacts (brown). (i) Donor KD (Inset: Cartoon representation showing secondary structure (Mg2+ (magenta sphere))) (ii) Receiver KD. (PDF) [file pcbi.1006796.s001.pdf]

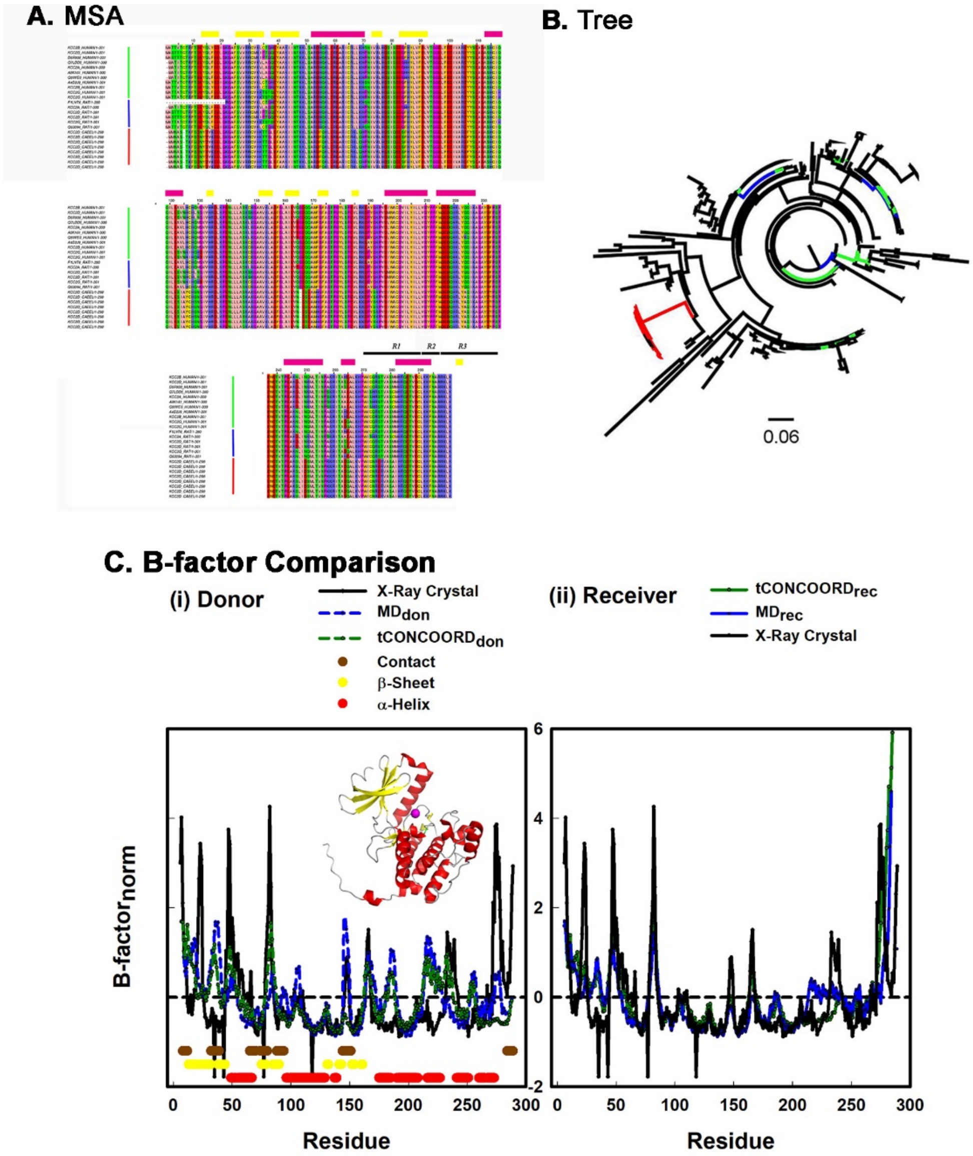

Figure S1 (related to Figure 1): CaMKII KD Conservation.
