# Supplementary figures and images for: Conformational coupling by trans-phosphorylation in calcium calmodulin dependent kinase II
Source: PLoS Comput Biol. 2019 May 31;15(5):e1006796. doi: 10.1371/journal.pcbi.1006796 (PMC6576796; doi:10.1371/journal.pcbi.1006796)

**A. Fold**

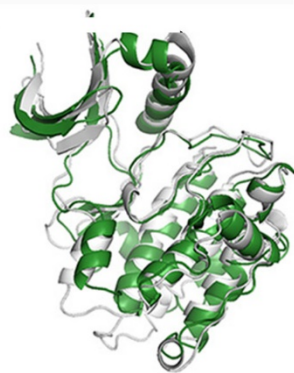

**B. Dynamics**

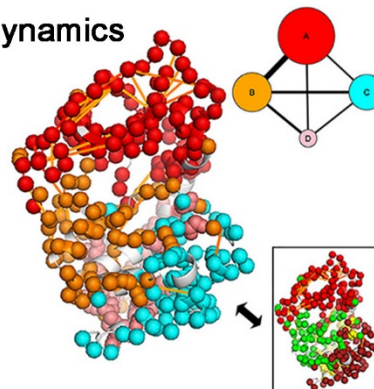

**Figure S2: PKA and CaMKII have Homologous Structure and Dynamics.**

Supplement: S2 Fig — A. Superposition of the C. elegans CaMKII KD (3KK8 [8]) with the open (1CMK [68]) form of PKA, a paradigm for analysis of EPKs, emphasize the canonical kinase fold. The structural homology is accompanied by sequence homology (E value < 10−5); but different quaternary structure. PKA is a tetramer with distinct catalytic and regulatory subunits, rather than a dodecamer with catalytic and regulatory domains in a single subunit as in CaMKII. PKA has an IQ motif for calmodulin binding and a flexible N-terminal helix upstream of this motif. The fit RMSD = 0.48 angstroms based on BLASTP multiple sequence alignment of 1CMK residues 41–312 with 3KK8 residues 11–286. B. PKA (1CMK) in the orientation shown in [26]. The community map obtained with tCONCOORD was consistent with, albeit coarser, than the published map derived from MD simulations [26] with four major communities of matching size and location. Smaller communities were not resolved probably because tCONCOORD does not account for anisotropic residue motions and solvent is implicit. Box: Community color coding as in [26]. (PDF) [file pcbi.1006796.s002.pdf]

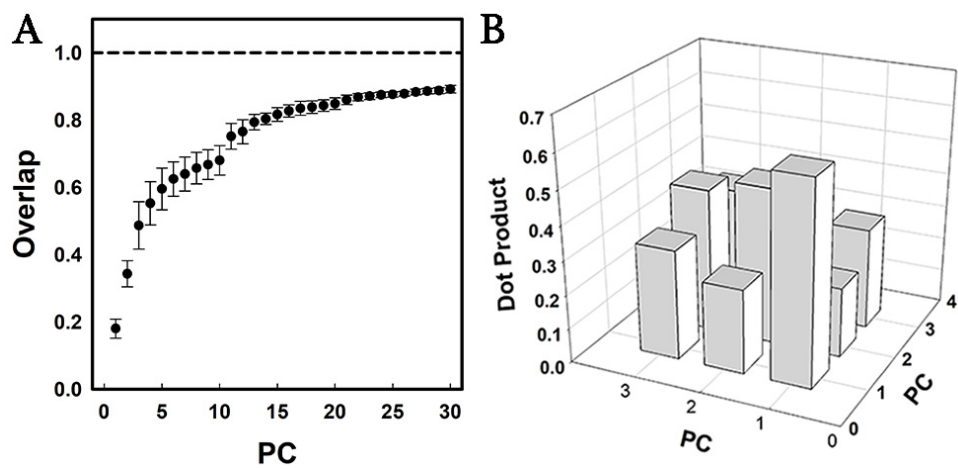

Figure S3: PCA eigenvector overlap

Supplement: S3 Fig — A. Cumulative overlap (root mean square inner product) [42] between pairs of the three 3KK8d_TPO replicas (mean (± se). B. Mean values for the 9 vector dot products between PCs 1–3 averaged over the 3 replicas. (PDF) [file pcbi.1006796.s003.pdf]

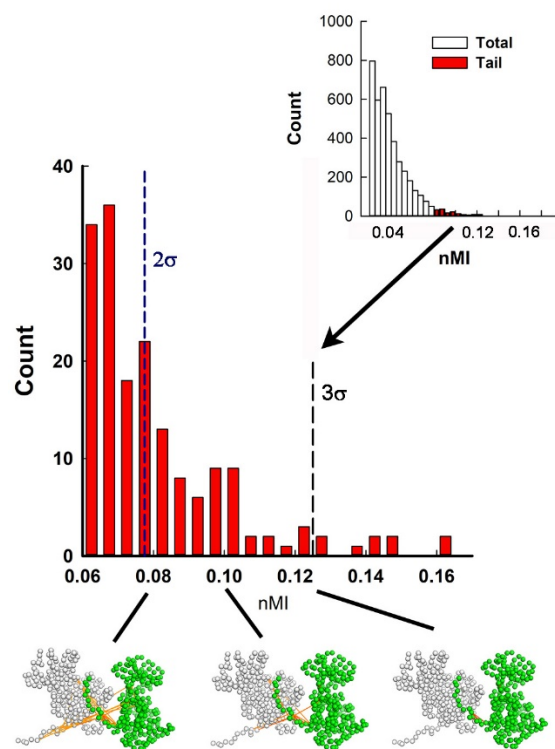

Figure S4: Selection of top couplings from the nMI distribution.

Supplement: S4 Fig — The tail (red) of the total (white) nMI distribution in the 2σ– 3σ range was analyzed for the top nMI couplings. Within this range formation of the R1-R1 relay dictated the choice of threshold (nMI = 0.09 (2.2 σ) for 3KK8d_TPO distribution shown). Snapshots at different thresholds (0.12 -> 0.10 -> 0.08) illustrate how the network forms starting with the initial central node. The selected nMI threshold for the 3KK8d-T distribution at similar significance level (2.2 σ) was 0.06. (PDF) [file pcbi.1006796.s004.pdf]

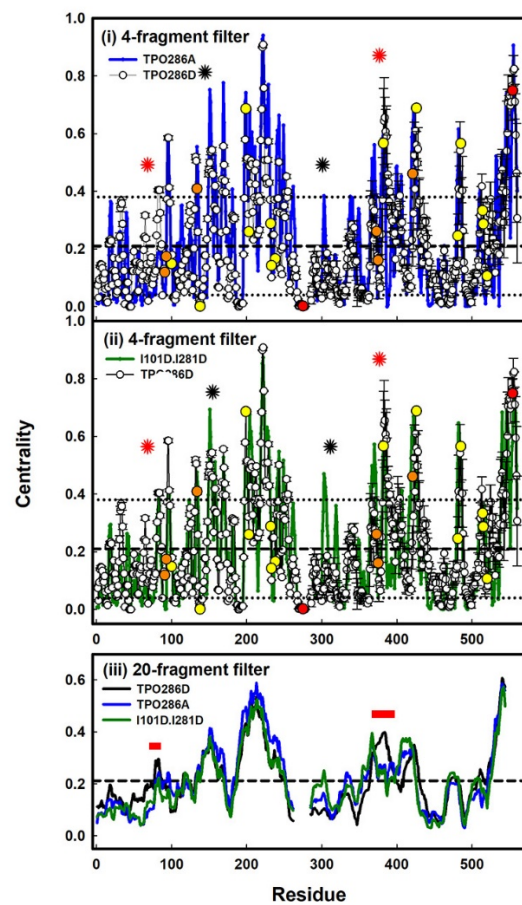

Figure S5: Network centrality of inactive mutant dimers

Supplement: S5 Fig — Comparison against 3 ensembles of the phospho-mimic TPO286D (open symbols, black line; error bars (se)). Ensemble size = (128^2). (i) TPO286A (blue). (ii) I101D.I281D. Circles represent S, T and phosphorylation sites as in Fig 2A. Asterisks indicate upshifts (red) and downshifts (black) in the phospho-mimic relative to the mutants. Lines denote means ± σ (dashed ± dotted). (iii) Running filtration of 20-fragments isolates two sequence segments (fragments 41–125; fragments 375–415) whose centrality is strengthened by activation (red bars). Pearson correlations (TPO286D–TPO286A; TPO286D-I101D.I281D; TPO286A-I101D.I281D) are 0.86, 0.89, 0.95 (Total), 0.55, 0.73, 0.88 (41–125) and -0.2, 0.34, 0.68 (375–415) respectively. (PDF) [file pcbi.1006796.s005.pdf]

**A. Transverse ( $H < 2$ )**

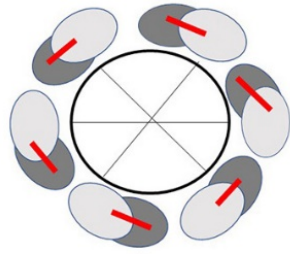

**B. Lateral ( $H > 2$ )**

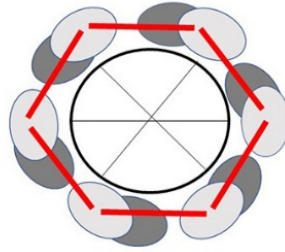

**Figure S6: Conformational coupling in the CaMKII holoenzyme.**

Supplement: S6 Fig — KDs in upper (light grey) and lower (dark grey) rings hexamer rings arrayed the central hub (white circle). Dashes (red) indicate couplings (number = n). A. Paired dimers form but do not interact. In absence of other interactions, H < 2. B. Subunits in each ring are conformationally coupled. H values > 2, and R1 dependent formation of KD multimers in solution [7, 9] are more straightforwardly reconciled with lateral conformational spread. (PDF) [file pcbi.1006796.s006.pdf]
