# Supplementary material for: Conformational coupling by trans-phosphorylation in calcium calmodulin dependent kinase II
Source: PLoS Comput Biol. 2019 May 31;15(5):e1006796. doi: 10.1371/journal.pcbi.1006796 (PMC6576796; doi:10.1371/journal.pcbi.1006796)
Supplement: S1 Table — (PDF) [file pcbi.1006796.s007.pdf]

Supporting Information – Table

REAGENT or RESOURCE	SOURCE	IDENTIFIER
**Software and Algorithms**		
Modeller	*(70)*	*https:/salilab.org/modeller*
Swiss-PDB Viewer	*(71)*	*www.expasy.org/spdbv*
Gromacs 2016.2, 4.5.7	*(72)*	*http://www.gromacs.org/*
CCP4 version7		*http://www.ccp4.ac.uk*
Poisson-Boltzmann solver	*(65)*	*http://www.poissonboltzmann.org*
GSATOOLS	*(33)*	*http://pandinilab.org/gsatools.html*
Pymol		*http://pymol.org*
tCONCOORD	*(25)*	*http://wwwuser.gwdg.de/~dseelig/tconcoord.html*
PsiPred	*(73)*	*http://bioinf.cs.ucl.ac.uk/psipred*
MUSCLE	*(74)*	*https://www.ebi.ac.uk/Tools/msa/muscle/*
Jalview	*(75)*	*http://www.jalview.org/*
FastTree 2.1	*(76)*	*http://www.microbesonline.org/fasttree/*
igraph	*(69)*	*https://cran.r-project.org/web/packages/igraph/*
Cytoscape		*http://www.cytoscape.org*
**Databases**		
Sequence files	Uniprot	*http://www.uniprot.org/*
PDB files	Protein Data Bank	*https://www.rcsb.org/*

Table S1: Web Resources – Software and Databases
